# Unravelling the Skin Secretion Peptides of the Gliding Leaf Frog, *Agalychnis spurrelli* (Hylidae)

**DOI:** 10.3390/biom9110667

**Published:** 2019-10-30

**Authors:** Carolina Proaño-Bolaños, Ailín Blasco-Zúñiga, José Rafael Almeida, Lei Wang, Miguel Angel Llumiquinga, Miryan Rivera, Mei Zhou, Tianbao Chen, Chris Shaw

**Affiliations:** 1 Natural Drug Discovery Group, School of Pharmacy, Queen’s University, Belfast 97 Lisburn Road, Belfast BT9 7BL, Northern Ireland, UK; 2 Biomolecules Discovery Group, Laboratory of Molecular Biology and Biochemistry, Universidad Regional Amazónica Ikiam, km 7 ½ vía Muyuna, Tena 150150, Ecuador; 3 Laboratorio de Investigación en Citogenética y Biomoléculas de Anfibios (LICBA), Centro de Investigación para la Salud en América Latina (CISeAL), Pontificia Universidad Católica del Ecuador, Av 12 de Octubre 1076 y Roca, Quito 170150, Ecuador

**Keywords:** dermaseptins, phylloseptins, frog skin secretion, peptidomics, tandem mass spectrometry, *Agalychnis spurrelli*

## Abstract

Frog skin secretions contain medically-valuable molecules, which are useful for the discovery of new biopharmaceuticals. The peptide profile of the skin secretion of *Agalychnis spurrelli* has not been investigated; therefore, the structural and biological characterization of its compounds signify an inestimable opportunity to acquire new biologically-active chemical scaffolds. In this work, skin secretion from this amphibian was analysed by molecular cloning and tandem mass spectrometry. Although the extent of this work was not exhaustive, eleven skin secretion peptides belonging to five peptide families were identified. Among these, we report the occurrence of two phyllokinins, and one medusin-SP which were previously reported in other related species. In addition, eight novel peptides were identified, including four dermaseptins, DRS-SP2 to DRS-SP5, one phylloseptin-SP1, and three orphan peptides. Phylloseptin-SP1 and dermaseptins-SP2 were identified in HPLC fractions based on their molecular masses determined by MALDI-TOF MS. Among the antimicrobial peptides, dermaseptin-SP2 was the most potent, inhibiting *Escherichia coli*, *Staphylococcus aureus,* and ORSA with a minimum inhibitory concentration (MIC) of 2.68 μM, and *Candida albicans* with an MIC of 10.71 μM, without haemolytic effects. The peptides described in this study represent but a superficial glance at the considerable structural diversity of bioactive peptides produced in the skin secretion of *A. spurrelli*.

## 1. Introduction

The resistance of microorganisms to current chemotherapy has limited successful treatments which stresses the urgent need for more efficient therapeutic strategies [1]. Animal venoms and secretions, such as the skin secretions of frogs, represent a treasure trove of small molecules that offer a promising way to tackle this global challenge [2]. The skin secretions of phyllomedusine hylids are complex molecular libraries composed by alkaloids, biogenic amides, inorganic molecules, opioids, steroids, nucleic acids, and proteins, and are mainly rich in a promising array of diverse peptides [3,4].

During the last 30 years, a large number of studies have reported the isolation, and biochemical and pharmacological characterization of frog peptides. Nearly 163 peptides have been described from 19 of 60 species of Phyllomedusinae. The most studied genus is *Phyllomedusa*, for which 13 species have revealed 227 peptides [4]. The next is *Agalychnis,* with five species analysed and 33 peptides identified; followed by *Cruziohyla,* with 33 peptides identified in one species; and *Phasmahyla,* for which the only species studied has revealed 23 peptides [5,6,7,8]. A comprehensive study of the skin secretion peptides of *Agalychnis spurrelli*, a frog found from southern Costa Rica to central-western Ecuador, remains to be performed.

From the structural point of view, these peptides showed a valuable chemical diversity of molecular architectures, from short molecules with few amino acids and a simple secondary structural element, such as temporins [9], to protease inhibitor peptides with tridimensional structures more complex and classified in several types [5]. Consequently, these variable scaffolds render them versatile compounds and potentially useful for the treatment of several diseases [5]. Peptides from frog skin present a wide repertoire of biological activities; for example, dermaseptins B2 and B3 have antitumor potential [10], [D4k]ascaphin-8 is anti-inflammatory candidate [11], and magainin-AM2 has been proposed for the diabetes therapy [12].

Representatives of several peptide families have been reported from *Agalychnis* skin secretions, such as dermaseptins, phylloseptins, and medusins. Dermaseptins are the most numerous peptides reported and these contain a W (Trp) residue in position three and the conserved sequence—AA(A/G)KAAL(G/N)A, in the mid region. The following dermaseptins have been reported: DRS-A3 to DRS-A5 of the blue-sided leaf frog, *A. annae*; DRS-C1 to DRS-C3 of the red-eyed tree frog, *A. callidryas*; DRS-DA2 to DRS-DA8 of the Mexican giant tree frog, *A. dacnicolor* (former *Pachymedusa dacnicolor*); DRS-L1 of the lemur leaf frog, *A. lemur* (former *Hylomantis*); and DRS-SP1 (insulinotropic peptide) of *A. spurrelli* (formerly *A. litodryas*) [13,14,15,16,17,18]. These peptides have a broad spectrum of cytolytic activity; i.e., DRS-DA5 inhibits the growth of *Escherischia coli*, *Bacillus subtilis* AIA, *Salmonella* STM 14028, and *Pseudomonas aeruginosa*. DRS-DA6, in contrast, is only active against *E. coli*, and DRS-DA7 is inactive against all of those microorganisms [19]. DRS-L1 showed high potency against *E. coli*. In addition, DRS-L1 was selectively cytolytic against red blood cells in contrast to HepG2 cells—a human liver cancer cell line [20]. Moreover, DRS-SP1 stimulated release of insulin from BRIN-BD11 cells—an insulin-secreting cell line [18].

Only one phylloseptin (PLS-L2) has been reported from *A. lemur* to date. Phylloseptins are antimicrobial peptides that contain the sequence FLS at their N-terminals, an H (His) in position 7, and C-terminal amidation. PLS-L2 has the unusual activity of releasing insulin from BRIN-BD11 cell lines [21].

Medusins are highly conserved antimicrobial peptides with only two variable positions: L/V at position 7 and A/S at position 14. They contain 19 residues and share the N-terminal sequence LLGMIP, and the C-terminal sequence LSKLamide. In *Agalychnis*, three medusins have been reported: medusin AC of *A. callidryas*, medusin-PD of *A. dacnicolor*, and medusin-AL (misidentified as Phylloseptin L1) of *A. lemur*. The sequence of medusin-AL is 100% identical to medusin-AC. Both medusins were active against *Staphylococcus aureus* and *Candida albicans*, without a haemolytic effect at their minimum inhibitory concentrations (MICs) [22]. Moreover, medusin-AL (same sequence as medusin-AC) was active against the chytrid fungus *Batrachochytrium dendrobatidis*, and it showed a similar cytolytic activity against erythrocytes and HepG2 cells at its lowest concentrations [20].

There have been significant advances in peptide sequencing, brought about by combining the techniques of tandem mass spectrometry (with data-dependent acquisition) and molecular cloning (which generates the peptide databases). In addition, the ability to prepare cDNA libraries from RNA isolated from skin secretions, rather than from the skin glands themselves, is a less invasive approach [23,24,25]. Integrating these biochemical techniques has helped with gaining deeper insights into the spectrum of peptides in complex samples [19,26,27,28]. In this context, this work aimed to identify and characterize the primary structures of peptides from the skin secretion of *Agalychnis spurrelli* by integrating proteomic and molecular tools, and subsequently, to evaluate their antimicrobial activity.

## 2. Materials and Methods

### 2.1. Animals and Skin Secretion Samples

Six specimens of *Agalychnis spurrelli* were employed in this study: two wild adults, collected in northwestern Ecuador and four captive-bred juveniles, provided by Centro Jambatu for Research and Conservation of Amphibians, in Ecuador. Skin secretions were stimulated by massaging the frogs’ backs, and then the whitish secretions were washed off the animals with distilled water. Samples were immediately frozen and stored at −20 °C. The wild frogs were returned to their habitat after the extraction and captive frogs were returned to their enclosures. The samples were freeze-dried, and later transported to Queen’s University, Belfast, for analysis. Collection and exportation permits were issued by the Ministry of Environment of Ecuador (MAE) as detailed in Acknowledgements.

### 2.2. Molecular Cloning

Five milligrams of lyophilized skin secretions were dissolved in 1 mL of cell lysis/binding buffer, and the polyadenylated mRNA was isolated using magnetic Dynabeads Oligo (dTs), as described by the manufacturer (Dynal Biotec, Oslo, Norway). A cDNA library was constructed and was subjected to 3′-rapid amplification of cDNA by using the SMART-RACE kit (Clontech, Mountain View, CA, USA). In brief, three sets of 3′-RACE reactions were employed, and each one used a nested universal primer (NUP), provided with the kit, and one of the following sense primers: S1: 5′-CAGCACTTTCTGAATTACAAGACCAA-3′, designed based on the signal sequence of phylloseptin-s5 of *Phyllomedusa sauvagii*; S2: 5′-GACCAAAGATGTCWTTCTTGAAGAAAT-3′, designed based on the opioid peptide of *A. dacnicolor*; or F2: 5′-TGAAGGGCCCTAACATGTCTT-3′, designed based on the signal sequence of dermaseptin-As1 of *A. spurrelli*. 3′-RACE products were purified and cloned using a pGEM-T vector system (Promega Corporation), and then sequenced using an ABI 3100 automated sequencer [23,24].

### 2.3. Reverse-Phase, High Performance Liquid Chromatography (HPLC) Fractionation

Five milligrams of freeze-dried skin secretion of *A. spurrelli* were dissolved in 1.2 mL of buffer A (99.95% H_2_O, 0.05% trifluoroacetic acid) and clarified by centrifugation. Supernatant (1 mL) was subjected to reverse phase HPLC employing a Waters Binary pump HPLC system fitted with an analytical column (Phenomenex Jupiter C-18; 4.6 × 250 mm). Peptides were eluted with a linear gradient formed from 100% buffer A (99.95% H_2_O, 0.05% trifluoroacetic acid) to 100% buffer B (80.00% Acetonitrile, 19.95% H_2_O, 0.05% trifluoroacetic acid) for 240 min at a flow rate of 1 mL/min. Fractions (1 mL) were collected each minute. Absorbance at 214 nm and 280 nm was monitored continuously.

### 2.4. Tandem Mass Spectrometry 

Twenty microliters of the remaining skin secretion samples, dissolved in buffer A from the HPLC step, were subjected directly to an analytical column (Phenomenex C-18; 4.6 × 150 mm) connected to an LCQ Fleet ESI ion-trap mass spectrometer (Thermo Fisher, San Jose, CA, USA). The linear elution gradient was formed from 100% buffer A (99.90% H_2_O, 0.1% formic acid) to 100% buffer B (19.9% H_2_O, 80% acetonitrile, 0.1% formic acid) for 135 min at a flow rate of 20 µL/min. Mass analysis was performed in a positive ion mode, and the conditions set for the spectra acquisition were a mass range of *m*/*z* 500–2000 and a relative intensity <50%. The parameters for electrospray ionization ion-trap mass spectrometry (ESI/MS) were the following: the spray voltage was +4.5 kV, the drying gas temperature was 320 °C, the drying gas flow was 200 µL/min, and the maximum accumulation time for the ion trap was 350 ms. Each fraction was analysed by two rounds of mass spectrometry (MS/MS) in order to obtain the amino acid sequences of the different peptides. Sequences obtained by MS/MS were compared with the sequence database built by molecular cloning in order to confirm their primary structures. The LCQ was controlled by Xcalibur software (Thermo, San Jose, CA, USA), and data analysis was performed using Proteome Discoverer 1.0 (Thermo, San Jose, CA, USA).

### 2.5. Bioinformatic Analysis

Nucleotide sequences were analysed by MEGA 6.0 and Vector NTI and compared with databases from the National Centre for Biotechnology Information (NCBI) by employing the BLAST tool [29,30,31]. Signal peptides were predicted using the SignalP 4.1 server, and theoretical peptide masses were calculated with peptide mass calculator v3.2 [32,33]. Secondary structure prediction was performed using the SOPMA programme and the physicochemical properties of the peptides were calculated using HeliQuest Compu Parameters and the Peptide property calculator from Bachem [34,35,36]. Finally, structural prediction models were constructed with HeliQuest and SWISS-MODEL online software [37].

### 2.6. Antimicrobial Activities of the Novel Peptides Identified 

For the in vitro evaluation of antimicrobial potentials of new biochemical structures identified herein, five synthetic peptides were purchased from a commercial supplier (Biomatik). Approximately 100 mg of each peptide was synthetized by Fmoc technology. The purities of the amidated peptides were greater than 90%. The lyophilized peptides were kept at −20 °C and diluted in dimethyl sulfoxide (DMSO) before the antimicrobial assays, which were performed according to broth microdilution methodology detailed by Proaño et al. [7]. Minimal inhibitory concentration of the synthetic peptides was determined against the following Gram-positive bacteria: *Staphylococcus aureus* ATCC 25923 and *S. aureus* oxacillin resistant strain; Gram-negative bacteria: *Escherichia coli* ATCC 25922 and *Klebsiella pneumonia* clinical isolate; and the opportunistic pathogenic yeast *Candida albicans*. Briefly, peptides were dissolved in DMSO at the following concentrations: 512, 256, 128, 64, 32, 16, 8, 4, 2, 1 × 10^2^ mg/L. Each bacteria/yeast was cultured until reach log phase and then it was diluted to obtain 1 × 10^6^ CFU/mL for bacteria and 1 × 10^5^ CFU/mL for the yeast. Assays were prepared in 96 well sterile plates where 198 µL of each microorganism was challenged with 2 μL of the diluted peptides at the serial dilutions mentioned above. Negative control included 2 μL of DMSO instead of peptide and 198 μL of microbial culture where we expected no inhibition. Another control included 200 μL of Muller Hinton broth without microorganisms to be used as blank with no growth. Seven replicates per peptide concentration was employed and experiments were performed in triplicates. Plates were cultured at 37 °C for 18 h and the inhibition of microbial growth was measured by spectrophotometry at 600 nm using a GloMax Multi Detection System (Promega, Madison, WI, USA). MIC was determined as the minimal concentration of peptide required to inhibit the growth of >95% of microbial growth.

### 2.7. The Determination of Toxicity to Red Blood Cells 

Aiming to determine the toxicity of frog peptides to red blood cells, in vitro assays were performed with different concentrations of each peptide. The diluted peptides (employing the same concentrations than for the antimicrobial assay described above) were incubated with a suspension of red blood cells (2%) (7.7 × 10^6^ ± 0.3 × 10^6^ cells/mL) at a ratio of 1:1 (*v*/*v*), in a total volume of 400 µL for 2 h at 37 °C. After the incubation period, the cells were centrifuged at 3000 rpm for 5 min and transferred to a 96 well plates. The haemolytic activity was monitored at 550 nm and was performed in triplicate. PBS and 2% (*v*/*v*) Triton X-100 were used as negative and positive controls, respectively. The concentrations that produce 100% haemolysis are reported.

## 3. Results

### 3.1. Molecular Cloning of the Novel Peptide Precursors Encoding cDNA Sequences, and the HPLC Profile

**Figure 1:**
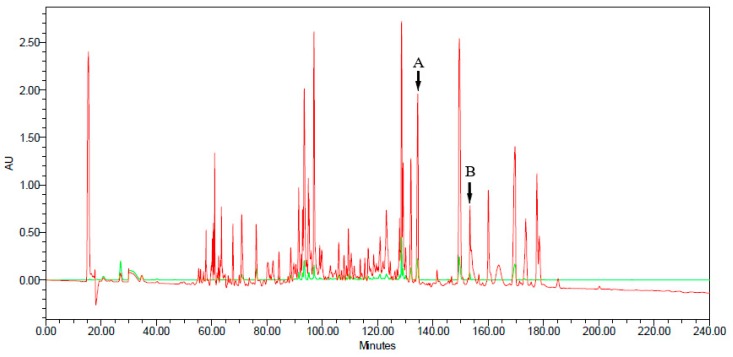
Reverse phase HPLC elution profile of skin secretion from *Agalychnis spurrelli* run over 240 min. Detection at 214 nm (red line); detection at 280 nm (green line). Letters A and B indicate the peak containing dermaseptin-SP2 and peak containing phylloseptin-SP1, respectively.

Eleven nucleotide sequences encoding peptide precursors were successfully cloned from the skin secretion of *Agalychnis spurrelli*. Among these were two phyllokinins and one medusin that were all reported previously in other species. The other eight mature peptide sequences were novel, including one phylloseptin, four dermaseptins, and three unclassified peptides. Nucleotide sequences were submitted to GenBank (NCBI) under accession numbers: MK532479-MK5232483 and MK766838-MK766843. The C18 HPLC profile of skin secretion of *Agalychnis spurrelli* revealed the presence of many peaks (Figure 1). The fractions containing dermaseptin-SP2 and phylloseptin-SP1 are identified with the letters A and B in the chromatogram, respectively. Herein we describe the main findings of each peptide family, with emphasis on novel primary structures of dermaseptins and phylloseptin, identified in this sample.

#### 3.1.1. Phyllokinins

Two 300 bp novel nucleotide sequences of peptide precursors that encoded phyllokinins were identified (accession numbers: MK766838 and MK766839). The translated open reading frames consisted of 186–189 nucleotides that encoded: (1) a 22 amino acid putative signal peptide; (2) a 29 amino acid acidic spacer that contained two pro-peptide processing sites (KR) and a sequence of four amino acids (ESPD or ESPE) at the C-terminal side of the latter; and (3) an 11 amino acid mature peptide (Supplementary Figure S1). These phyllokinin were named [Ser^6^, Val^10^, Asp^11^]-phyllokinin and [Thr^6^, Val^10^, Asp^11^]-phyllokinin to highlight their similar but different amino acid sequences.

The nucleotide sequences of [Ser^6^, Val^10^, Asp^11^]-phyllokinin and [Thr^6^, Val^10^, Asp^11^]-phyllokinin were 87%–91% similar to the preprophyllokinin-HA1 of *Phyllomedusa hypochondrialis*, the Hyp^3^-Thr^6^-BK precursor of *A. callidryas*, and the Thr^6^-phyllokinin of *P. sauvagii* (accession numbers AM283482.1, HE967330.1, and AJ549500.1 respectively). However, the comparison of the mature peptide sequences within the NCBI database using BLAST/p yielded different results; i.e., while the amino acid sequence of [Thr^6^, Val^10^, Asp^11^]-phyllokinin was, as expected, 100% identical to that of Thr^6^-BK of *A. dacnicolor* and Hyp^3^-Thr^6^-BK of *A. callidryas* (cannot confirm hydroxy-proline) (accession numbers CCJ67650.1 and CCJ67651.1 respectively), the sequence of [Ser^6^, Val^10^, Asp^11^]-phyllokinin was, in contrast, 100% identical to the bradykinin-like peptide RD-11 of *Ascaphus truei* and vespakinin-T of *Vespa tropica* wasp venom (accession numbers P84824.1 and ADD13606.1 respectively) (Supplementary Table S1).

#### 3.1.2. Medusin-AS

A nucleotide precursor of 433 pb encoding a medusin was identified in the skin (accession number MK766840). The open reading frame consisted of: (1) a 22 amino acid putative signal peptide; (2) a 27 amino acid acidic spacer that contained two KR pro-peptide processing sites; (3) an 18 amino acid mature medusin sequence; and (4) a glycine amide donor residue (Supplementary Figure S2). This peptide precursor was named medusin-AS to represent its origin in *A. spurrelli*.

The medusin-AS nucleotide precursor sequence was 95% similar to medusin-AC of *A. callidryas* (accession number HE863819.1). In addition, the translated amino acid sequence of the mature medusin-AS was 100% identical to the medusin-AC (accession number CCI17382.1), and 95% similar to medusin-PD of *A. dacnicolor*, medusin-PH of *P. hypochondrialis*, and medusin-PS of *Phyllomedusa sauvagii* (accession numbers: CCI79381.1, CCI79383.1, and CAP17494.1 respectively) (Supplementary Table S2).

#### 3.1.3. Phylloseptin-AS1

**Figure 2:**
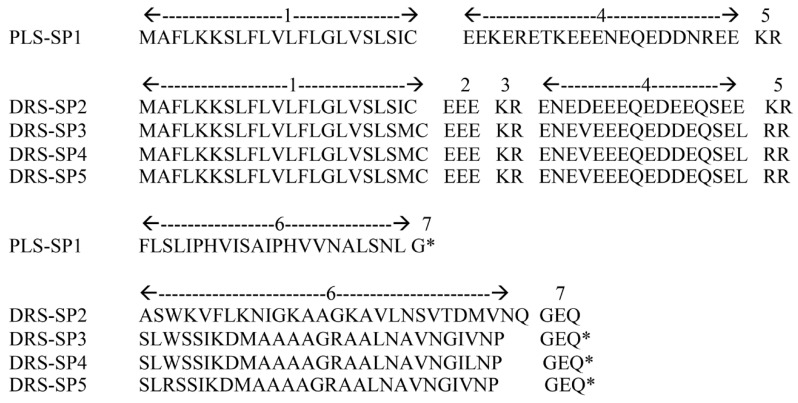
Translated open reading frame amino acid sequences of phylloseptin-SP1 (PLS-SP1) and dermaseptin-SP precursors (DMS-SP2-SP5). Precursor domain structures: (**1**) Putative signal peptides. (**2**,**4**) Acidic spacers. (**3**,**5**) Dibasic propeptide convertase processing sites. (**6**) Mature peptides. 7 Glycine amide donors.

A novel nucleotide sequence encoding a phylloseptin was cloned from the skin secretion (accession number MK532479). The translated open reading frame consisted of: (1) a 22 amino acid putative signal peptide; (2) a 23 amino acid acidic spacer with one pro-peptide processing site; (3) a 22 amino acid mature peptide; and (4) a glycine residue amide donor at the C-terminus (Figure 2 and Supplementary Figure S3). This peptide was named phylloseptin-SP1—SP for the species name “*spurrelli*”—in accordance with the nomenclature rules proposed for the antimicrobial peptides of Phyllomedusine frogs [38].

**Figure 3:**
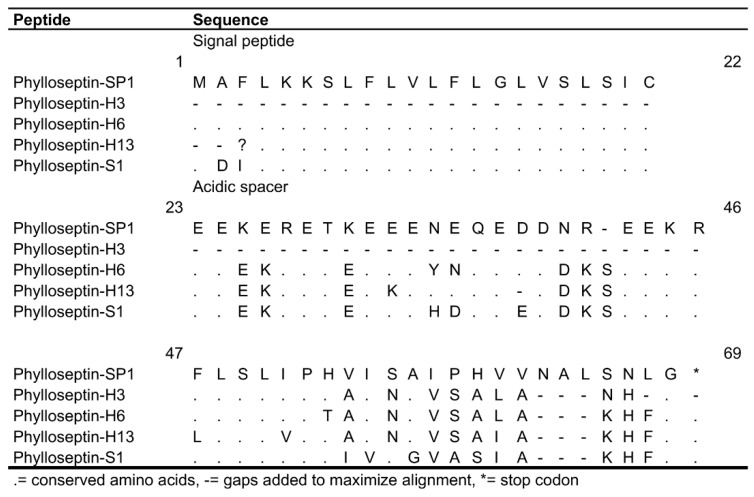
Comparison of amino acid sequence similarity of phylloseptins. PLS-SP1 of *A. spurrelli*, PLS-H3, PLS-H6, and PLS-H13 of *Phyllomedusa hypochondrialis*, and PLS-S1 of *P. sauvagii* (accession numbers: P84568.1, Q0VZ41.1, AM292541.1, and FN667968.1 respectively).

The nucleotide sequence of the precursor of phylloseptin-SP1 was 82% similar to the precursor of phylloseptin-H13 and medusin-PH of *P. hypochondrialis*, medusin-PS (previously known as phylloseptin-S5), and phylloseptin-S1 of *P. sauvagii* (accession numbers: AM292541.1, HE863820.1, AM903081.1, and AM903077.1 respectively). However, the mature peptide sequence of phylloseptin-SP1 was only 62% similar to phylloseptin-H3 of *P. hypochondrialis* (accession number P84568.1) (Figure 3).

PLS-SP1 was identified in HPLC fractions 153 and 154 due to its monoisotopic molecular mass of 2354.00 as identified by MALDI-TOF mass spectrometric analysis. Later, the mass of this peptide was confirmed by an LCQ ESI MS full scan that showed ions +2 = *m*/*z* of 1177.58, +3 = *m*/*z* of 785.58, and +4 = *m*/*z* of 589.50 (Table 1 and Supplementary Figure S4).

#### 3.1.4. Dermaseptins

Four novel nucleotide sequences of precursors of 343–365 bp encoding dermaseptins were identified in the skin secretions (accession numbers: MK532480–MK532483). The translated open reading frames consisted of: (1) 22 amino acid putative signal peptides; (2) 23 amino acid acidic spacers that contained two pro-peptide processing sites KR and RR; (3) 27–28 amino acid mature peptide sequences; and (4) a tripeptide sequence GEQ at the C-terminus, where glycine acts as an amide donor (Figure 2 and Supplementary Figure S3).

**Figure 4:**
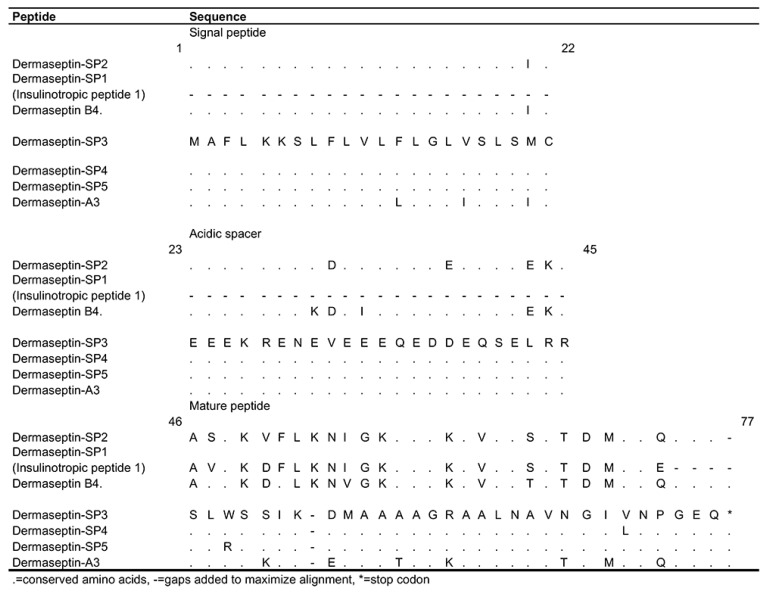
Comparison of amino acid sequence similarity of dermaseptins. DRS-SP1–DRS-SP4, and insulinotropic peptide 1 of *A. spurrelli*, DRS-A3 of *A. annae*, and DRS-B4 of *P. bicolor* (accession numbers: P86941.1, O93223.1, and P81486.1)**.**

These peptides were named dermaseptins-SP following current nomenclature rules, where SP states the first two letters of the species name *“spurrelli”* [38]. The nucleotide precursors of dermaseptins-SP3–SP5 were 88%–95% similar to the precursor of a number of dermaseptins, including the following: DRS-A3 of *A. annae*; DRS-C1 of *A. callidryas*; DRS-DA2 of *A. dacnicolor*; DRS-H1 and DRS-H2 of *P. hypochondrialis*; and DRS-B4 of *P. bicolor* (accession numbers: AJ004185.1, AM944842.1, AJ005190.1, AM229015.1, AM229016.1, and Y16565.1) [38]. However, the mature sequences of dermaseptins-SP3–SP5 showed lower similarity (65%–88%) with the dermaseptin-like peptide of *Schistosoma mansoni*, and the dermaseptins DRS-A3, DRS-C1, DRS-C3, DRS-H2, and DRS-H3 (accession numbers: P83914.1, O93223.1, CAQ16442.1, AAO62952.1, P84937.1, and Q17UY8.1). In contrast, the translated mature sequence of dermaseptin-SP2 shared 77%–89% similarity with the insulinotropic peptide 1 of *A. spurrelli* (here renamed as dermaseptin-SP1 or DRS-SP1), and the dermaseptins DRS-B4 of *P. bicolor*, DRS-S7 of *P. sauvagii*, and DRS-H1 and DRS-H2 of *P. hypochondrialis* (accession numbers P86941.1, P81486.1, CAD92230.1, CAJ76139.1, and CAJ76140.1 (Figure 4).

In addition, DRS-SP2 was identified in HPLC fraction 134. Its monoisotopic molecular mass of 2988.24 Da was identified by MALDI-TOF mass spectrometric analysis. The fraction also contained another peptide of 3005.36 Da. that co-eluted (Table 1 and Supplementary Figure S4).

#### 3.1.5. Orphan Peptides

Three novel precursors were identified and temporarily assigned to the orphan peptide group because their highly divergent sequences did not allow them to be assigned to any of the peptide families described to date (accession numbers: MK766841–MK766843). The first novel nucleotide sequence of peptide unknown-1 was 82%–93% similar to medusin precursors of *A. dacnicolor, A. callidryas, P. hypochondrialis*, and *P. sauvagii* (accession numbers HE863818.1, HE863819.1, HE863820.1, and AM903081.1). However, its translated mature sequence was highly divergent and did not produce any significant hits when compared with the databases in NCBI. In addition, the two novel nucleotide sequences of peptides unknown-2 and 3 were 88%–90% similar to tryptophyllin precursors, including tryptophyllin-PdT1 and PdT2 of *A. dacnicolor*, tryptophyllin-AcT2 of *A. callidryas*, and tryptophyllin HA5 of *P. hypochondrialis*. Peptides unknown-1 and 2 could be considered phenylalanine-rich peptides due to their high F content (43%). Nevertheless, their translated mature peptide sequences did not produce any relevant hits when they were compared with NCBI databases using BLAST-P.

### 3.2. Peptide Identification by Tandem Mass Spectrometry

The peptide database created by molecular cloning was compared with the database generated by fragmentation of the peptide ions in the tandem mass spectrometric analysis of the skin secretion of *A. spurrelli.* As a result, 10 putative mature peptides were 100% identified while one sequence was not found at all (Table 1). In summary, 15 to 103 peptide fragments were sequenced per peptide, which contained between 11 and 31 amino acids and a molecular mass range of between 1273.66 and 2988.62 Da.

### 3.3. The Antimicrobial and Haemolytic Activities of Synthetic Peptides Based on Amino Acid Sequences Identified and Toxicity to Red Blood Cells

The synthetic dermaseptins showed antimicrobial activity against Gram-positive and Gram-negative bacteria, and yeast. The peptides were more active against bacteria than yeast (Table 2). The dermaseptin-2 was the most potent peptide, inhibiting bacterial growth at 2.68 µM against *E. coli, S. aureus*, and a *S. aureus* oxacillin resistant strain [39,40,41,42].

**Figure 5:**
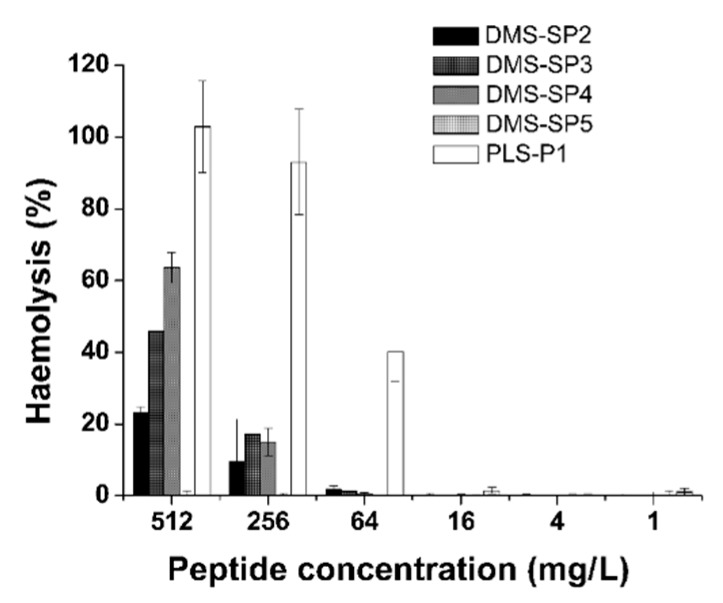
Haemolytic activity of Dermaseptins SP2–SP5 and Phylloseptin SP-1. The total hemolysis (100%) was determined using Triton X-100.

Dermaseptins induced no red blood cell lysis at MIC concentrations. In general, dermaseptins only triggered a significant haemolytic effect at concentrations higher than 512 µM (Figure 5). The phylloseptin-SP1 showed greater toxicity to red blood cells than did the dermaseptins, also increasing with increasing peptide concentration.

### 3.4. Secondary Structures and Physicochemical Properties of Synthetic Peptides

The net charge, hydrophobic moment, and α-helix parameters of synthetic peptides evaluated against bacteria and yeasts were determined in silico by use of bioinformatic tools (Supplementary Table S3; Table S4). Interestingly, the most active peptide, dermaseptin-2, possessed a higher net charge (+4) when compared to the other peptides tested.

**Figure 6:**
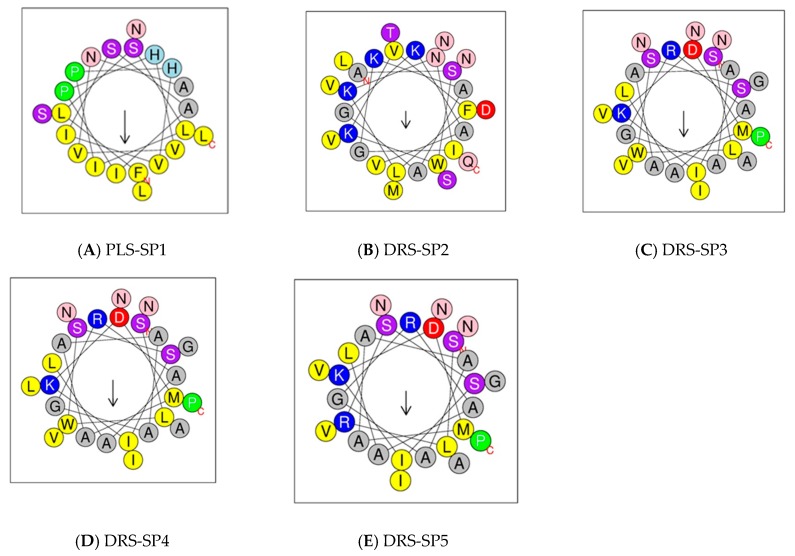
Helical wheel projections of the phylloseptins and dermaseptins-SP of *A. spurrelli*. Helical wheel projections of phylloseptin-SP1 (**A**), Dermaseptin-SP2 (**B**), Dermaseptin-SP3 (**C**), Dermaseptin-SP4 (**D**), and Dermaseptin-SP5 (**E**).

Bioinformatic analyses have revealed the high propensity of peptides to adopt an α-helical conformation. Dermaseptin-SP2 had a lower α-helix% (Supplementary Table S3; Table S4). In addition, helical wheel plots illustrate the amphipathic characteristics and properties of alpha helices in the analysed peptides (Figure 6).

## 4. Discussion

The granular glands of frogs are responsible for the production of diverse peptides, some of which have shown a broad spectrum of action with potential biomedical applications [2]. The HPLC chromatogram of a skin secretion of *A. spurrelli* confirmed the abundance of peptides present (Figure 1). Other biochemical studies have revealed similar molecular complexity and diversity from skin secretions of frogs [4]. By peptidomic analysis and integrating tandem mass spectrometry with molecular cloning, eleven peptides belonging to five families were identified in *A. spurrelli* skin secretion and screened for antimicrobial activity. The peptide sequences identified in this work are detailed and discussed in the light of their families.

### 4.1. Phyllokinins

The deduced amino acid sequences revealed the presence of two previously reported bradykinin-like peptides. This was confirmed by BLAST/p comparisons which showed that [Ser^6^, Val^10^, Asp^11^]-phyllokinin is identical to the bradykinin-like peptide RD-11 of the frog, *Ascaphus truei,* and vespakinin-T of the hornet, *Vespa tropica*; and that [Thr^6^, Val^10^, Asp^11^]-phyllokinin is identical to Hyp^3^-Thr^6^-BK of *A. callidryas* and Thr^6^-BK of *A. dacnicolor.* The nucleotide comparison of frog peptides precursors-encoding cDNA, ignoring the vespakinin-T precursor because it is too divergent, revealed that these related phyllokinin precursors have 24 non-synonymous substitutions that encode for 12 amino acids and eight synonymous substitutions in their signal and acidic spacer peptides. In addition, there are eight synonymous substitutions in the region corresponding to the mature peptides between these precursors. The occurrence of the same Thr^6^-phyllokinin in the three species of *Agalychnis* is not surprising and reflects taxonomic relationships. In contrast, the presence of the bradykinin-like peptide RD-11 of *A. truei* (also known as vespakinin-T) of the hornet, *V. tropica*, was unexpected. *Ascaphus truei* is an ancient basal species and sister-group of all other amphibian species. Therefore, this could show an early evolutionary origin of the ancestral gene of this bradykinin-like peptide, which should be confirmed whenever the precursor sequence of RD-11 becomes available (Supplementary Table S1) [43]. Here, we have named this peptide precursor as [Ser^6^, Val^10^, Asp^11^]-phyllokinin for the sake of consistency with other bradykinin-related peptides of phyllomedusine frogs. The occurrence of this bradykinin-like peptide (vespakinin-T) in the venom of the hornet, *V. tropica,* could be due to convergent evolution because its precursor is too divergent to be related to the frog phyllokinin precursors.

A previous study showed that RD-11 is able to induce relaxation of pre-contracted mouse trachea interacting with the bradykinin B2 receptor in the same way as bradykinin, only less potently [43,44]. Although the biological role of these bradykinin-related peptides remains unclear, the observation of antipredator behaviour in some snakes, after the initial taste of some frogs that contain these peptides, indicates a defensive role. In addition, studies that show bradykinin to be a potent stimulator of gastrointestinal smooth muscle and pain sensitivity pathways in mammals, as well as its effect on the vascular system of snakes, strongly suggest an antipredator-defensive role among these peptides [3,45,46].

### 4.2. Medusin-AS

A medusin with an identical amino acid sequence to medusin-AC was identified in this skin secretion. The nucleotide sequences of corresponding precursors are virtually identical, having three non-synonymous substitutions that lead to three different amino acids and four synonymous substitutions in the region corresponding to the signal peptide and acidic spacer. In addition, the mature peptide sequence contains only one synonymous substitution. It is notable that this medusin is highly conserved across species. At the moment, medusin-AC is known to occur in *A. callidryas*, *A. spurrelli*, *A. lemur* (described as Phylloseptin-L1), and in *Cruziohyla calcarifer*. (Supplementary Table S2). This is the first antimicrobial peptide occurring in four different species of two distinct genera and could be indicative of a secondary role acting on cell receptors. At the moment, this peptide has been found to have antimicrobial activity against *S. aureus*, *C. albicans*, and *Batrachochytrium dendrobatidis*, but has been found to be inactive against *E. coli*. In addition, it is cytolytic against HepG2 human hepatoma-derived cells and human erythrocytes at similar concentrations (LC_50_ 45 µM and LC_50_ 40 µM respectively) [20,22].

### 4.3. Phylloseptin-AS1

A novel phylloseptin was identified in the skin secretion of *A. spurrelli*. It shares limited similarity with other phylloseptins (63%), although the nucleotide precursor sequences showed higher similarity (82%) with phylloseptins and medusins, which indicates a close relationship between these two peptides families [22]. As shown in our findings, conserved amino acids are concentrated in their N-terminal regions, while the C-terminal end is highly divergent. As is the case for PLS-H3, H6, and S1, PLS-SP1 shares the N-terminal conserved sequence FLSLIP, an H in position seven, and a C-terminal amidated residue that was predicted from the position of the glycine amide donor (Figure 3). These are all the main characteristics of phylloseptins, so the new sequence was named phylloseptin AS-1 in accordance with the nomenclature proposed for antimicrobial peptides of this family [38]. In addition, PLS-SP1 constitutes the longest phylloseptin (with 22 residues) reported to date (Figure 2).

Additionally, phylloseptin-SP1 was identified in HPLC fractions 153 and 154 (Figure 1). The observed *m*/*z* of 2354.00 in those fractions was determined by MALDI-TOF mass spectrometry and confirmed later by tandem mass spectrometry as 2354.43, which was very close to the calculated mass of 2353.83 for this phylloseptin. (Supplementary Figure S3).

Cationic peptides, such as phylloseptins, usually exhibit antimicrobial activity that disrupts the bacterial cell membrane. The lysis starts by electrostatic interaction of the cationic peptides with the anionic microbial membrane, followed by the rearrangement of the peptides into an amphipathic helix. The alpha helix is inserted into the membrane through interaction of the hydrophobic face of the helix with the lipid acyl chains of the bilayer, causing disruption of the membrane after reaching a critical peptide concentration [40,47,48,49].

PLS-SP1 has similar physicochemical properties to PLS-S1, including a high hydrophobicity (0.848–0.806 H), hydrophobic moment (0.516–0.551 µM), and a very high alpha helix percentage (100%–94.7%) in its structure. However, PLS-SP1 and PLS-S1 have three main differences in terms of sequence, size, and charge. PLS-SP1 and PLS-S1 share only 42.11% sequence similarity, and the sequence of PLS-SP1 is three amino acids longer than PLS-S1 (22 versus 19 residues respectively). In addition, PLS-SP1 is less cationic than PLS-S1 (+1 versus +2 respectively). PLS-SP1 showed lower antimicrobial effect when compared to PLS-S1 and the other peptides tested, evidencing the essential role of charge to antimicrobial activity (Table 2). The effect of charge on the differential bactericidal effect was demonstrated in another study with PLS-S3, where the reduced cationicity (+1 instead of +2) was attributed as a main factor for its lack of antimicrobial activity, despite it having similar physicochemical properties to other paralogous antimicrobial phylloseptins (PLS-S1, PLS-S2, and PLS-4) (Supplementary Figure S3). The same effect was evidenced in the synthetic peptide P19(1|E) with a +1 charge that is devoid of antimicrobial activity but shares a similar mean hydrophobicity, amphipathicity, and degree of secondary structure formation to P19(3|E), P19(5), and P19(6) (with +3, +5 and +6 charges respectively). According to this paper, increasing the peptide charge to at least +3 is required for an antimicrobial activity in these peptides: [48].

In summary, PLS-SP1 is a hydrophobic and amphipathic helical peptide, which exhibited low antimicrobial activity at physiological pH, probably owing to its low charge (+1). However, its histidine content PLS-SP1 can convert it into an antimicrobial molecule at acidic pH by increasing cationicity. These hypotheses should be confirmed by appropriate experimental approaches in future studies.

### 4.4. Dermaseptin-SP

The following group of peptides includes four novel dermaseptins identified in the skin secretion of *A. spurrelli*. The novel peptides were named dermaseptin-SP2 to SP5 in accordance with the proposed nomenclature [38]. The similarity with nucleotides in other dermaseptin precursors was very high (88%–95%), indicating that the novel peptides belong to the dermaseptin superfamily.

The first novel dermaseptin-SP2 was 92% similar to the insulinotropic peptide-1 of *A. spurrelli* (formerly *A. litodryas*), and 85% similar to dermaseptin-B4 of *P. bicolor*. The insulinotropic peptide-1 (FSIP) belongs to the dermaseptin family because it contains a W (Trp) in the three position and the mid-region conserved sequence, but was not recognized as such in the corresponding publication nor in the peptide database of the NCBI. Therefore, hereafter this peptide is called dermaseptin-SP1 (DRS-SP1). The sequence alignment of DRS-SP2 with its closest paralogous peptide DRS-SP1 and the orthologous DRS-B4, showed that there are only three amino acid substitutions in the acidic spacer and six substitutions in the mature peptide region (Figure 4). The physical properties of these peptides are very similar in terms of hydrophobicity (0.317–0.358 H), hydrophobic moment (0.275–0.308), and helicity (78.57%–100%). However, they differ in cationicity, where DRS-SP2 is highly cationic +4, followed by DRS-B4 +3 and DRS-SP1 +1 (Supplementary Figure S4). The effect of cationicity over biological activity was demonstrated with phylloseptin-S3 and the synthetic peptide P19(1|E) [40,48]. In brief, cationicity is directly proportional to antimicrobial activity and cytotoxicity in amphipathic helical peptides.

**Figure 7:**
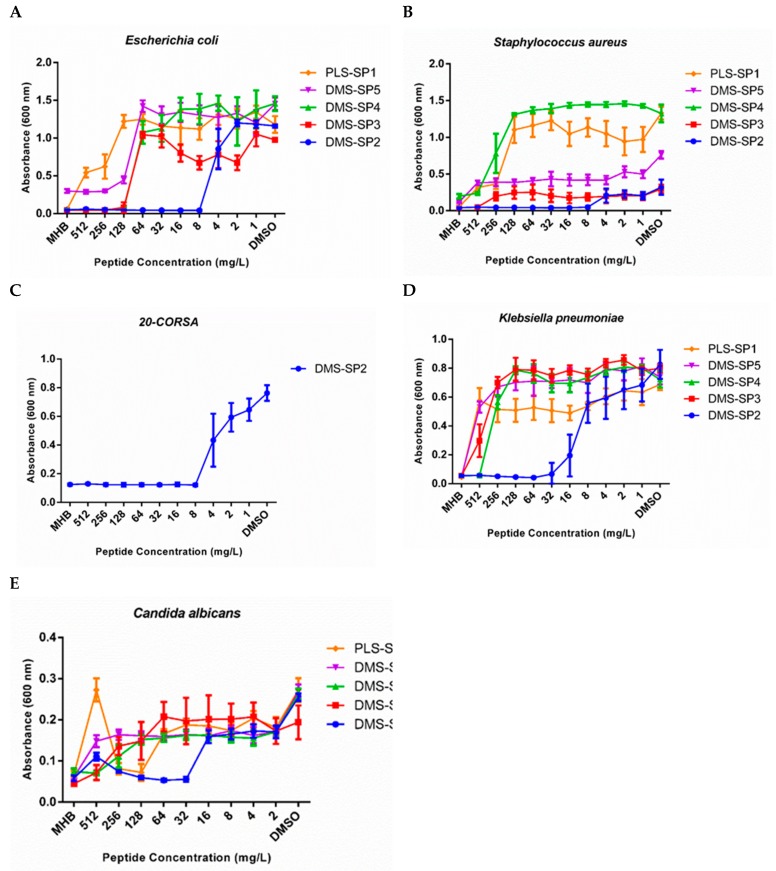
Inhibition effects of peptides against (**A**) *E. coli*, (**B**) *S. aureus*, (**C**), *S. aureus* Oxacillin resistant (**D**) *Klebsiella pneumonia*, and (**E**) *C. albicans*. PLS-SP1 stands for phylloseptin-SP1. DMS-SP2 is dermaseptin-SP2, DMS-SP3 is dermaseptin-SP3, DMS-SP4 is dermaseptin-SP4, and DMS-SP5 is dermaseptin-SP5.

In this sense, DRS-B4, with +3 charge, showed a potent antimicrobial activity against *S. aureus* (MIC 3 µM), *P. aeruginosa* (MIC 4.6–11.6 µM), and *E. coli* (MIC 0.5–2.3 µM), but it also exhibited cytotoxicity at 12 µM [39]. Continuing this trend, DRS-SP2 was the most potent antimicrobial peptide (MIC 2.68 µM against *E. coli. S. aureus*, and ORSA (Figure 7). This synthetic molecule shares structural features with others AMPs, such as hydrophobicity and net charge, reflecting the positive amino acids of primary structure [6]. These features are important for molecular mechanism of action of peptides [36]. Several studies have showed the cell membrane as the main target of antibacterial peptides [50,51]. Briefly, cationic and hydrophobic peptides trigger leakage of bacterial content or osmotic shock by opening artificial water channels or membrane destabilization [52]. The variable effect on bacteria and yeast can be a result from a difference in membrane composition [36]. Interestingly, the dermaseptin-2 peptide with higher activity has the higher charge, which can be favour electrostatic interactions with negative elements of microbial membrane. Basically, the killing mechanisms of bacteria induced by cationic peptides is based on membranolytic activity; however, their antifungal properties derive from biochemical and molecular processes more complex, which require the entry of them into the cell in the most cases. The antifungal peptides exert their effects by several mechanisms, such as interaction with intracellular targets; cell wall binding; the inhibition of DNA, RNA, and protein syntheses; the induction of signalling cascades or metabolic pathways; and the production of free radicals [53,54]. The dichotomy between toxicity to human cell and microbial cells is key to development of peptide drugs. In light of this, dermaseptins induced the death of bacteria and yeast without haemolytic effect at MIC concentrations.

The other three novel dermaseptin sequences, DRS-SP3 to SP5, were similar to dermaseptins DRS-A3, DRS-C1, DRS-C3, DRS-H2, and DRS-H3 of *A. annae*, *A. callidryas*, and *P. hypochondrialis* respectively. As expected, the amino acid sequences between the paralogous peptides DRS-SP3, -SP4, and SP-5 are 100% identical in the region corresponding to the signal peptide and acidic spacer, and 96% identical in the mature peptide. There are only two variable positions in the mature sequence of these dermaseptins—in position three (W/R) and position 25 (V/L). In comparison with the closest related orthologous peptide DRS-A3, there are three amino acids substitutions in the signal peptide and seven in the mature peptide (74% similarity). The antimicrobial activity against *E. coli* of DMS-SP3 to DMS-SP5 was lower than DMS-SP2 (47.50–96.06 µM versus 2.68 µM) but comparable with the MIC of Dermaseptin-S1 (46 µM) of *Phyllomedusa sauvagii*. Moreover, DMS-SP’s MIC was, as excepted, a lot higher the MIC of a conventional antibiotic, such as ampicillin (6 µM against *E. coli* and 0.1 µM against *S. aureus*)

### 4.5. Orphan Peptides

The last three novel peptides have similarity in their precursors with medusins and tryptophyllin precursors, but their highly divergent mature sequences do not permit more precise identification. Therefore, they have been temporarily assigned to the orphan peptide family until further information becomes available. This highlights that there is still much to discover in the complexity of amphibian skin secretions.

### 4.6. Tandem Mass Sequencing

Ten of the thirteen sequences analysed above were 100% identified by tandem mass spectrometry, which shows the robustness of the combined proteomic/molecular cloning approach in revealing novel peptides sequences in previously unanalysed species for which there is limited information. Notably, two of the three sequences that were unable to be identified by this methodology belong to the tryptophyllins, and this could be attributed to either (1) low expression of these peptides under the detection limit of the instrument and method employed; (2) no expression, implying that these tryptophyllins were transcribed but not translated by the skin glands at the time-point the samples were collected; or (3) that these peptides were subject to rapid degradation.

In conclusion, 11 novel peptides from *A. spurrelli* were identified and confirmed by molecular cloning and peptidomic approaches, which required a minimum amount of sample without harming the animal source. These peptides belong to five distinct peptide families. Dermaseptins, mainly dersmaseptin-2, showed a promising antimicrobial effect, which can be explored for translation in antibacterial therapies. This study stresses the remarkable variety of skin secretion peptides in *Agalychnis spurrelli*, and encourages further analysis in this and other unexplored amphibian species.

## Figures and Tables

**Table 1:** Peptides with sequences confirmed by LCQ MS/MS sequencing.

Peptide	Precursor Sequence	Identify by	Coverage %	# Peptides Fragments	# AAs	LCQ MW [Da]	Theoretical MW [Da]	Score
[Ser6, Val10, Asp11)-phyllokinin	RPPGFSPFRVD	mc,ms2	100	19	11	1273.66	1274.44	13.80
[Thr6, Val10, Asp11)-phyllokinin	RPPGFTPFRVD	mc,ms2	100	15	11	1287.67	1288.47	8.08
Medusin-AS	LLGMIPLAISAISALSKL-NH2	mc,ms2	100	50	19	1810.10	1809.12	77.05
Phylloseptin-SP1	FLSLIPHVISAIPHVVNALSNL-NH2	mc,ms2	100	60	23	2353.35	2352.37	59.59
Dermaseptin-SP2	ASWKVFLKNIGKAAGKAVLNSVTDMVNQ-NH2	mc,ms2	100	83	31	2988.62	2987.64	32.49
Dermaseptin-SP3	SLWSSIKDMAAAAGRAALNAVNGIVNP-NH2	mc,ms2	100	103	30	2696.41	2695.42	47.66
Dermaseptin-SP4	SLWSSIKDMAAAAGRAALNAVNGILNP-NH2	mc,ms2	100	90	30	2710.42	2709.44	13.3
Dermaseptin-SP5	SLRSSIKDMAAAAGRAALNAVNGIVNP-NH2	mc,ms2	100	75	30	2666.43	2665.44	14,13
Orphan peptide SP-1	IIGMIPDLISMISKL-NH2	mc,ms2	100	84	17	1642.94	1641.96	108.95
.rphan peptide SP-2	FLFLPFIGRRK	mc,ms2	100	31	11	1392.84	1393.74	22.05
Orphan peptide SP-3	FLLPPFFGRKK	mc	0	*	11	*	1349.69	*

* = not found, mc = molecular cloning, ms^2^ = tandem mass spectrometry.

**Table 2:** Minimal inhibitory concentrations (MICs) of synthetic dermaseptins and phylloseptin-SP1 of *Agalychnis spurrelli.*

Synthetic Peptide.	Sequence	MIC μM (mg/L)					
		*E. coli* ATCC 25922	*S. aureus* ATCC 25923	*S. aureus* Oxacillin Resistant (ORSA Strain 20)	*K. pneumoniae* Clinical Isolate	*C. albicans*	Accession Number
Dermaseptin-SP2	ASWKVFLKNIGKAAGKAVLNSVTDMVNQ-NH2	2.68 (8) *	2.68 (8) *	2.68 (8)	10.71 (32)	10.71 (32) *	MK532480
Dermaseptin-SP3	SLWSSIKDMAAAAGRAALNAVNGIVNP-NH2	47.50 (128) *	189.98 (512) *	ND	>189.98 (>512)	>189.98 (>512) *	MK532481
Dermaseptin-SP4	SLWSSIKDMAAAAGRAALNAVNGILNP-NH2	47.25 (128) *	189.00 (512) *	ND	189.00 (512)	>189.00 (>512) *	MK532482
Dermaseptin-SP5	SLRSSIKDMAAAAGRAALNAVNGIVNP-NH2	96.06 (256) *	>192.12 (>512) *	ND	>189.00 (>512)	384.24 (1024) *	MK532483
Dermaseptin-B4	ALWKDILKNVGKAAGKAVLNTVTDMVNQ-NH2	5.00 **	3.00 **	ND	ND	ND	P81486
Dermaseptin-S1	ALWKTMLKKLGTMALHAGKAALGAAADTISQGTQ	46.00 **	60.00 **	ND	ND	ND	AJ564794.1
Phyloseptin-SP1	FLSLIPHVISAIPHVVNALSNL-NH2	>217.69 (>512)	>217.69 (>512)	ND	>189.00 (>512)	>217.69 (>512)	MK532479
Phyloseptin-S1	FLSLIPHIVSGVASIAKHFG	70.00 ***	6.25 ***	ND	ND	ND	AM903077.1
Ampicillin		6.0 ****	0.1 ****				
Dermaseptin-SP2	ASWKVFLKNIGKAAGKAVLNSVTDMVNQ-NH2	2.68 (8) *	2.68 (8) *	2.68 (8)	10.71 (32)	10.71 (32) *	MK532480

ND = not determined, * Cuesta et al., 2019 (41), ** Charpantier, et al., 1998 (39), *** Raja et al., 2013 (40), **** Reimer et al., 1981 (42).

## Supplementary Materials

The following are available online at: https://www.mdpi.com/2218-273X/9/11/667/s1: Figure S1: Nucleotide and translated open reading frame sequences of phyllokinin precursors from *Agalychnis spurrelli*. Figure S2: Nucleotide and translated open reading frame sequence of medusin-AS precursor. Figure S3: Nucleotide and translated open reading frame amino acid sequences of phylloseptin and dermaseptin-SP precursors. Putative signal peptides are double underlined, mature peptides are single underlined, and stop codons are indicated by asterisks. Figure S4: Mass spectral analysis of HPLC fractions 153 and 154 containing phylloseptin-SP1. (A) Spectrum denotes a peak of *m*/*z* 2354.00 by MALDI-TOF MS. (B) LCQ MS ESI full scan denotes ions 2+ *m*/*z* 1177.58, 3+ *m*/*z* 785.58, and +4 *m*/*z* 589.50 corresponding to PLS-SP1. Figure S5: Mass spectral analysis of antimicrobial HPLC fraction 134 containing dermaseptin-SP2. Spectrum denotes a main peak of m/z 2988.24 by MALDI-TOF MS. Table S1: Alignment of precursor nucleotide sequences and translated open reading frames of phyllokinins-As1 and As2 of *Agalychnis spurrelli*. Table S2: Alignment of nucleotide and translated open reading frame amino acid sequences of medusin-AS of *A. spurrelli* and medusin-AC of *A. callidryas*. Table S3: Physicochemical properties of phylloseptin-SP1 and other related phylloseptins. PLS-SP1 of *A. spurrelli*; PLS-H3, PLS-H6, and PLS-H13 of *P. hypochondrialis*; and PLS-S1 of *P. sauvagii*. Table S4: Physicochemical properties of dermaseptins-SP and other related dermaseptins. DRS-SP1 to DRS-SP4, and insulinotropic peptide 1 of *A. spurrelli*; DRS-A3 of *A. annae*; and DRS-B4 of *P. bicolor*.
