# Supplementary material for: Unravelling the Skin Secretion Peptides of the Gliding Leaf Frog, Agalychnis spurrelli (Hylidae)
Source: Biomolecules. 2019 Oct 30;9(11):667. doi: 10.3390/biom9110667 (PMC6920962; doi:10.3390/biom9110667)
Supplement: Supplementary file 1 [file biomolecules-09-00667-s001.zip › Supplementary Figure 1.docx]

A)

M S F L K K S L

1 AGCACTTTCT GAATAACAAG ACCAAACATG TCTTTCTTGA AGAAATCTCT

· L L V L F L G L V S F S I C E E E ·

51 TCTCCTGGTA CTTTTCCTTG GATTGGTTTC CTTTTCCATC TGTGAGGAAG

· K R E T E E E E N E D D M D E E

101 AGAAAAGAGA GACTGAAGAG GAGGAGAATG AAGATGACAT GGACGAAGAA

S E E K K R E S P D R P P G F S P ·

151 AGTGAAGAGA AAAAGAGAGA GTCTCCAGAT AGACCTCCCG GTTTCAGTCC

· F R V D *

201 TTTTCGAGTT GATTAACACA TTGAGAAAAT GTAACGTGAT AATCTAAGGA

251 GCATAATTAT CAGTAATTAT GCCAAAAACA TATTAAAGCA TATTTAACGG

301 AAAAAAAAAA AAAAAAAAAA AAAAAAA

B)

M S F L K K S L

1 CAGCACTTTC TGAATTACAA GACCAAACAT GTCTTTCTTG AAGAAATCTC

· F L V L F L G L V S F S I C E E

51 TTTTCTTGGT ACTTTTCCTT GGATTGGTTT CCTTTTCCAT CTGTGAAGAA

E K R E T E E E E N E D E M N E E ·

101 GAGAAAAGAG AGACTGAAGA AGAGGAGAAT GAAGATGAAA TGAACGAAGA

· S E E K R E S P E R P P G F T P F ·

151 AAGTGAAGAG AAGAGAGAGT CTCCAGAGAG ACCTCCTGGT TTCACTCCTT

· R V D *

201 TTCGAGTTGA TTAACACATT GAAAAAAATG TAACATGCGG TAATCTAAGG

251 AGCACAATTA TCAGTAATTA TGCTAAAAAC ATATTAAAAC ATATTTAACA

301 AAAAAAAAAA AAAAAAAAAA AAAAAAA

C)

🡨------------------1---------------🡪 2 3 🡨---------------4-------------🡪 5

[Ser^6^, Val^10^, Asp^11^]-Phyllokinin MSFLKKSLLLVLFLGLVSFSIC EEE KR ETEEEENEDDMDEESEEK KR

[Thr^6^, Val^10^, Asp^11^]-Phyllokinin MSFLKKSLFLVLFLGLVSFSIC EEE KR ETEEEENEDEMNEESEE KR

🡨6🡪 🡨--------7--------🡪

[Ser^6^, Val^10^, Asp^11^]-Phyllokinin ESPD RPPGFSPFRVD*

[Thr^6^, Val^10^, Asp^11^]-Phyllokinin ESPE RPPGFTPFRVD*

Supplementary Figure 1. Nucleotide and translated open reading frame sequences of phyllokinin precursors from *Agalychnis spurrelli*. Putative signal sequences are double-underlined, mature peptide sequences are single underlined and stop codons are indicated by asterisks. A) [Ser^6^, Val^10^, Asp^11^]-Phyllokinin. B) [Thr^6^, Val^10^, Asp^11^]-Phyllokinin. C) Domain structures of phyllokinin precursors: 1. Putative signal peptide. 2,4. Acidic spacers. 3, 5. Dibasic propeptide convertase processing sites. 7. Mature peptides.
