# Supplementary material for: Unravelling the Skin Secretion Peptides of the Gliding Leaf Frog, Agalychnis spurrelli (Hylidae)
Source: Biomolecules. 2019 Oct 30;9(11):667. doi: 10.3390/biom9110667 (PMC6920962; doi:10.3390/biom9110667)
Supplement: Supplementary file 1 [file biomolecules-09-00667-s001.zip › Supplementary Figure 2.docx]

A)

M A F L K K S L F ·

1 AGCACTTTCA GAAGATCGAC CAACATGGCT TTCCTGAAGA AATCTCTTTT

· L V L F L G L V S L S V C E E E K ·

51 TCTTGTACTA TTCCTTGGAT TAGTTTCCCT TTCCGTCTGC GAAGAAGAGA

· R E S E E E K N E Q E E D D R D

101 AAAGAGAGAG TGAAGAGGAA AAAAATGAGC AAGAGGAAGA CGATCGTGAT

E R S E E K R L L G M I P L A I S ·

151 GAGAGAAGTG AAGAGAAGAG ATTGTTGGGC ATGATACCAC TGGCAATATC

· A I S A L S K L G *

201 TGCAATATCT GCACTTTCAA AACTAGGTTA ATAAAATGTA AACTTTCATA

251 ACTTTAAGGG CCCATCTACA CGGCATGGTG TTTCGTCTGG TTTTCTGGAT

301 GGTTTGCAGA TTTTTTTGCC GGAATTTGTC CATTGCCGTG TGGACCTGGC

351 CAAAGGAGTA CAATTATCAA TAATTGTCCG AAAAATATAT TAAAGCATAT

401 TTAACCAACA AAAAAAAAAA AAAAAAAAAA AAA

B)

🡨------------------1-----------------🡪 2 3 🡨----------------4---------------🡪 5

Medusin-AS MAFLKKSLFLVLFLGLVSLSVC EEE KR ESEEEKNEQEEDDRDERSEE KR

🡨-------------6------------🡪 7

Medusin-AS LLGMIPLAISAISALSKL G*

Supplementary Figure 2. Nucleotide and translated open reading frame sequence of medusin-AS precursor. A) Putative signal peptide is double underlined, the mature peptide is single underlined and the stop codon is indicated by an asterisk. B) Domain structure of medusin-AS precursor. 1. Putative signal peptide. 2, 4. Acidic spacers. 3, 5 Dibasic propeptide processing sites. 6. Mature peptide. 7. Glycine amide donor.
