# Supplementary material for: Unravelling the Skin Secretion Peptides of the Gliding Leaf Frog, Agalychnis spurrelli (Hylidae)
Source: Biomolecules. 2019 Oct 30;9(11):667. doi: 10.3390/biom9110667 (PMC6920962; doi:10.3390/biom9110667)
Supplement: Supplementary file 1 [file biomolecules-09-00667-s001.zip › Supplementary Figure_3.docx]

1. Phylloseptin-SP1

M A F L K K S L F ·

1 GCACTTTCTG AAGAAAGACC AAACATGGCT TTCCTGAAGA AATCTCTTTT

· L V L F L G L V S L S I C E E K E ·

51 CCTTGTACTT TTCCTTGGAT TAGTTTCCCT TTCAATCTGT GAAGAAAAGG

· R E T K E E E N E Q E D D N R E

101 AAAGAGAGAC TAAAGAGGAA GAAAATGAGC AAGAGGACGA CAATCGTGAA

E K R F L S L I P H V I S A I P H ·

151 GAGAAGAGAT TCTTAAGCTT GATACCACAT GTAATATCTG CAATACCACA

· V V N A L S N L G *

201 TGTAGTAAAT GCACTTTCAA ACTTGGGTTA ATAAAATATA ACATTTCATA

251 CTTCTAAGGA GCACAATTAT CGATGATTGT CCCCAAAATA TATTAAAGCA

301 TATTTAAACA AAAAAAAAAA AAAAAAAAAA AAAAAAAA

1. Dermaseptin-SP2

M A F L K K S L F ·

1 GCACTTTCTG AATACAAGAC CAACATGGCT TTCCTGAAGA AATCTCTTTT

· L V L F L G L V S L S I C E E E K ·

51 CCTTGTACTA TTCCTTGGAT TGGTCTCTCT TTCTATCTGT GAAGAAGAGA

· R E N E D E E E Q E D E E Q S E

101 AAAGAGAAAA TGAAGATGAG GAGGAACAGG AAGATGAGGA GCAAAGTGAA

E K R A S W K V F L K N I G K A A ·

151 GAGAAGAGAG CATCATGGAA AGTTTTTTTA AAAAATATAG GAAAAGCAGC

· G K A V L N S V T D M V N Q G E Q ·

201 AGGAAAAGCG GTTTTAAATT CAGTTACGGA TATGGTAAAT CAAGGAGAGC

· *

251 AATAAAGCAA ACAAATGTAA AATCAATAAT TGTGCCAGCG CTACATTAAA

301 GCATTTTGAA CAAAAAAAAA AAAAAAAAAA AAAAAAAAAA AAA

C) Dermaseptin-SP3

M A F L K K S L F ·

1 AGCACTTTCT GAATAAAGAC CAACATGGCT TTCCTGAAGA AATCTCTATT

· L V L F L G L V S L S M C E E E K ·

51 CCTTGTACTA TTCCTTGGAT TGGTCTCTCT TTCTATGTGT GAAGAAGAGA

· R E N E V E E E Q E D D E Q S E

101 AAAGAGAAAA TGAAGTTGAG GAGGAACAAG AAGATGACGA GCAAAGTGAA

L R R S L W S S I K D M A A A A G ·

151 TTGAGGAGAT CACTGTGGAG TTCAATAAAA GACATGGCAG CAGCTGCAGG

· R A A L N A V N G I V N P G E Q * ·

201 AAGAGCGGCT TTAAATGCAG TTAATGGTAT AGTAAATCCG GGAGAGCAAT

251 AAAGTTAAGA AAATGTAAAA TCAAATTGCT TTAAGGAGTA CAATTATGAA

301 TAATTGTGCT CATCATAAAT AAACCAAATT TAACTGAACA AAAAAAAAAA

351 AAAAAAAAAA AAAAA

1. Dermaseptin-SP4

M A F L K K S L F ·

1 AGCACTTTCT GAAAACGGAC CAACATGGCT TTCCTGAAGA AATCTCTATT

· L V L F L G L V S L S M C E E E K ·

51 CCTTGTACTA TTCCTTGGAT TGGTCTCTCT TTCTATGTGT GAAGAAGAGA

· R E N E V E E E Q E D D E Q S E

101 AAAGAGAAAA TGAAGTTGAG GAGGAACAAG AAGATGACGA GCAAAGTGAA

L R R S L W S S I K D M A A A A G ·

151 TTGAGGAGAT CACTGTGGAG TTCAATAAAA GACATGGCAG CGGCTGCAGG

· R A A L N A V N G I L N P G E Q * ·

201 AAGAGCGGCT TTAAATGCAG TTAATGGTAT ATTAAATCCG GGAGAGCAAT

251 AAAGTTAAGA AAATGTAAAA TCAAATTGCT TTAAGGAGTA CAATTATGAA

301 TAATTGTGCC TATCATAAAT AAACCAAATT TAACAAAAAA AAAAAAAAAA

351 AAAAAAAAAA A

1. Dermaseptin-SP5

M A F L K K S L F ·

1 GCACTTTCTG AAAACAAGAC CAACATGGCT TTCCTGAAGA AATCTCTATT

· L V L F L G L V S L S M C E E E K ·

51 CCTTGTACTA TTCCTTGGAT TGGTCTCTCT TTCTATGTGT GAAGAAGAGA

· R E N E V E E E Q E D D E Q S E

101 AAAGAGAAAA TGAAGTTGAG GAGGAACAAG AAGATGACGA GCAAAGTGAA

L R R S L R S S I K D M A A A A G ·

151 TTGAGGAGAT CACTGCGGAG TTCAATAAAA GACATGGCAG CAGCTGCAGG

· R A A L N A V N G I V N P G E Q * ·

201 AAGAGCGGCT TTAAATGCAG TTAATGGTAT AGTAAATCCG GGAGAGCAAT

251 AAAGTTAAGA AAATGTAAAA TCAAATTGCT TTAAGGAGTA CAATTATGAA

301 TAATTGTGCA AATCATAAAT AAACCAAATT TAACAAAAAA AAAAAAAAAA

351 AAAAAAAAAA A

Supplementary Figure 3. Nucleotide and translated open reading frame amino acid sequences of phylloseptin and dermaseptin-SP precursors. Putative signal peptides are double underlined, mature peptides are single underlined and stop codons are indicated by asterisks.
