# Supplementary material for: Unravelling the Skin Secretion Peptides of the Gliding Leaf Frog, Agalychnis spurrelli (Hylidae)
Source: Biomolecules. 2019 Oct 30;9(11):667. doi: 10.3390/biom9110667 (PMC6920962; doi:10.3390/biom9110667)
Supplement: Supplementary file 1 [file biomolecules-09-00667-s001.zip › Supplementary Figure_4.docx]

**A)**

**
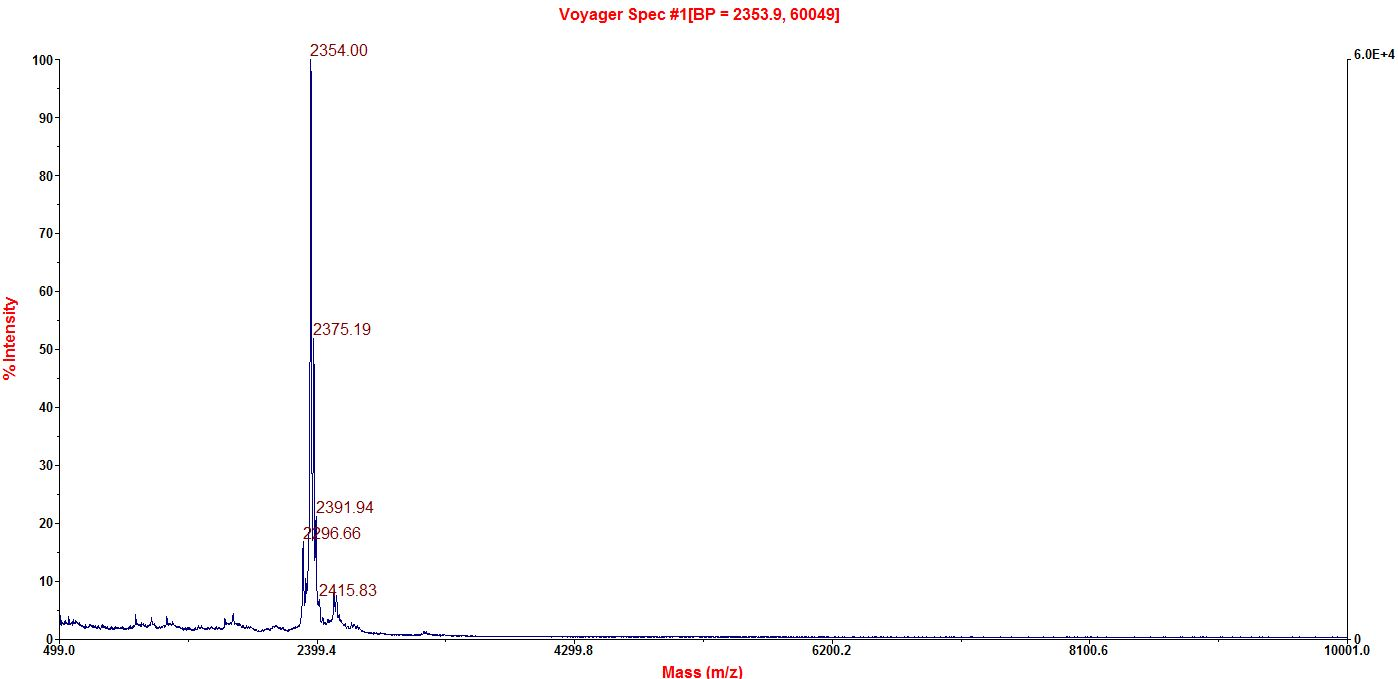
**

**B)**

**
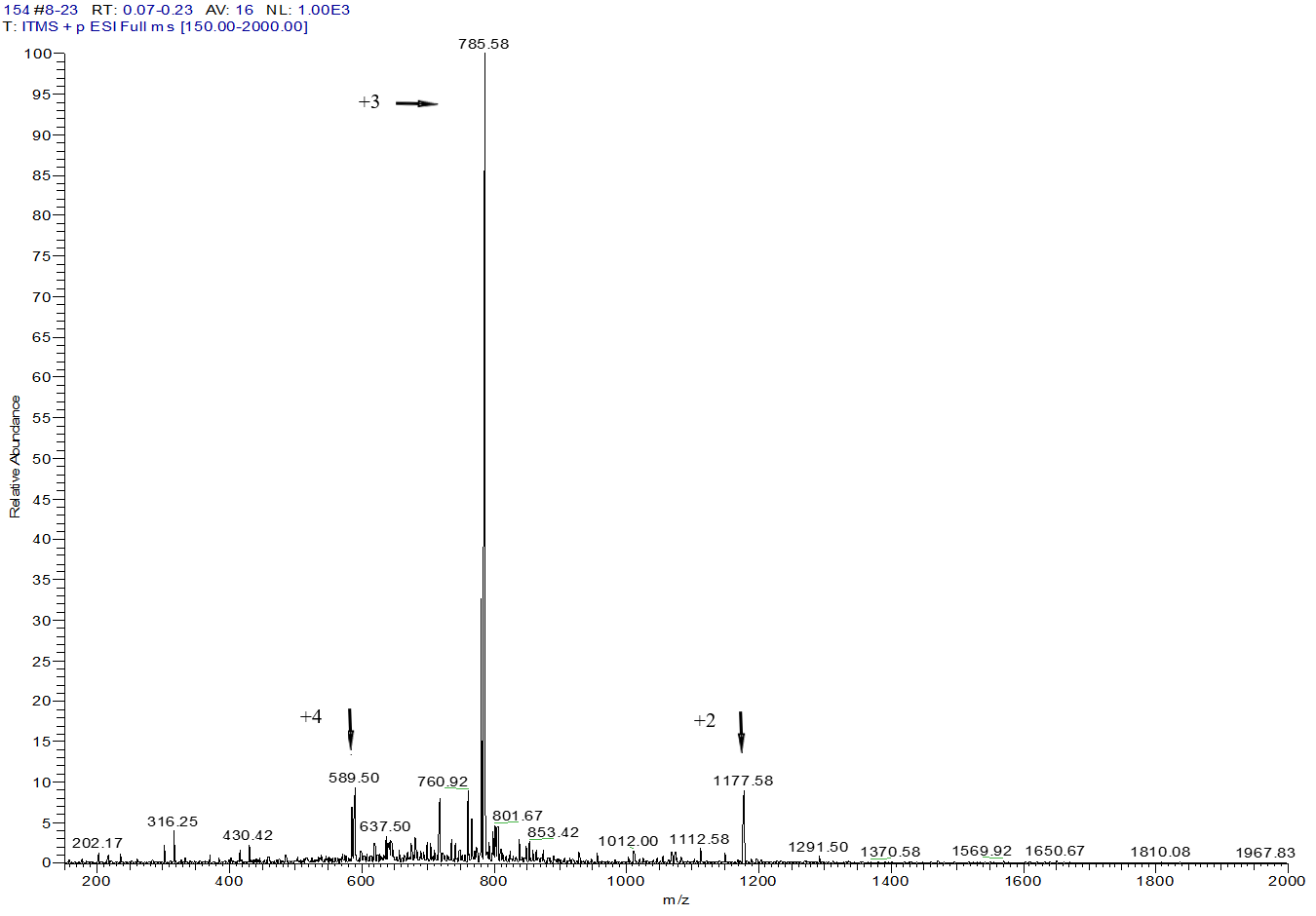
**

Supplementary Figure 4. Mass spectral analysis of HPLC fractions 153 and 154 containing phylloseptin-SP1. A) Spectrum denotes a peak of m/z 2354.00 by MALDI-TOF MS. B) LCQ MS ESI full scan denote ions 2+ m/z 1177.58, 3+ m/z 785.58, and +4 m/z 589.50 corresponding to PLS-SP1.
