# Supplementary material for: Unravelling the Skin Secretion Peptides of the Gliding Leaf Frog, Agalychnis spurrelli (Hylidae)
Source: Biomolecules. 2019 Oct 30;9(11):667. doi: 10.3390/biom9110667 (PMC6920962; doi:10.3390/biom9110667)
Supplement: Supplementary file 1 [file biomolecules-09-00667-s001.zip › Supplementary Figure_5.docx]

**
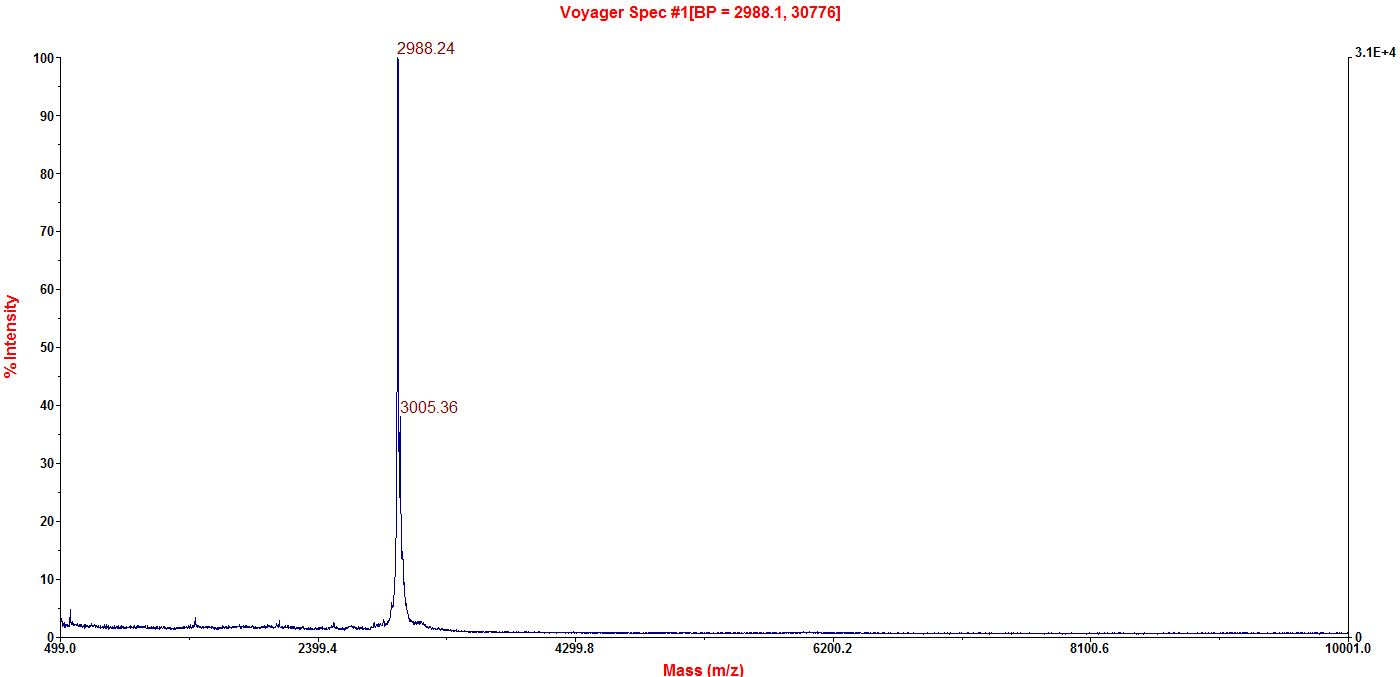
**

**Supplementary Figure 5**. Mass spectral analysis of antimicrobial HPLC fraction 134 containing dermaseptin-SP2. Spectrum denotes a main peak of m/z 2988.24 by MALDI-TOF MS.
